# Effects of Whole-Body Vibration on Ankle Control and Walking Speed in Individuals with Incomplete Spinal Cord Injury

**DOI:** 10.3390/brainsci15040405

**Published:** 2025-04-17

**Authors:** Jasmine M. Hope, Anastasia Zarkou, Cazmon Suri, Edelle C. Field-Fote

**Affiliations:** 1 Division of Physical Therapy, School of Medicine, Emory University, Atlanta, GA 30322, USA; jhope@emory.edu; 2 Spinal Cord Injury Research Laboratory, Virginia C. Crawford Research Institute, Shepherd Center, Atlanta, GA 30309, USA; anastasia.zarkou@shepherd.org (A.Z.);; 3 Program in Applied Physiology, School of Biological Sciences, Georgia Institute of Technology, Atlanta, GA 30318, USA

**Keywords:** foot drop, spinal reflex circuit, spasticity, spinal cord injury, walking, whole-body vibration, dorsiflexion

## Abstract

Background/Objectives: After spinal cord injury (SCI), poor dorsiflexor control and involuntary plantar-flexor contraction impair walking. As whole-body vibration (WBV) improves voluntary muscle activation and modulates reflex excitability, it may improve ankle control. In this study, the dosage effects of WBV on walking speed, dorsiflexion, and spinal reflex excitability were examined. Methods: Sixteen people with chronic motor-incomplete SCI participated in this randomized sham-control wash-in study. Two weeks of sham stimulation (wash-in phase) were followed by either 2 weeks of eight repetitions (short bout) or sixteen repetitions of WBV (long bout; intervention phase) per session. Walking speed, ankle angle at mid-swing, and low-frequency depression of the soleus H-reflex were measured before and after the wash-in phase and before and after the intervention phase. Results: A significant dosage effect of WBV was not observed on any of the measures of interest. There were no between-phase or within-phase differences in ankle angle during the swing phase or in low-frequency depression. When dosage groups were pooled together, there was a significant change in walking speed during the intervention phase (mean = 0.04 m/s, standard deviation = 0.06, *p* = 0.02). There was not a significant correlation between overall change in walking speed and dorsiflexion angle or low-frequency depression during the study. Conclusions: Whole-body vibration did not have a dosage-dependent effect on dorsiflexion during the swing phase or on spinal reflex excitability. Future studies assessing the role of corticospinal tract (CST) descending drive on increased dorsiflexor ability and walking speed are warranted.

## 1. Introduction

One of the most obvious consequences of spinal cord injury (SCI) is the loss or impairment of walking ability. For this reason, it is not surprising that regaining the ability to walk is a top priority for persons with SCI [1]. An important component of walking that is frequently impaired after SCI is the ability to lift the forefoot during the swing phase [2]. This inability to achieve adequate dorsiflexion, known clinically as foot drop, can be further hindered by the involuntary muscle contractions and stiffness associated with spasticity in the plantar flexors. To optimize functional independence and improve quality of life in persons with SCI, it is important to understand the neurophysiological mechanisms that contribute to these deficits in ankle control and how they respond to interventions that modulate neural excitability.

The volitional control of the dorsiflexors is more highly dependent on corticospinal drive than other lower extremity muscles [3,4]. Foot drop is a result of disrupted descending drive from the corticospinal tract (CST) to the tibialis anterior (TA) due to damage to the spinal cord. Additionally, upper motor neuron damage can lead to spasticity in the plantar flexors, which is associated with a reduced ability to activate the dorsiflexors and adds to the impairment of ankle control during walking [5]. In persons with SCI, interventions that either increase the descending drive to the TA or decrease spasticity in the soleus are associated with improved walking function [5].

In persons with SCI, whole-body vibration (WBV) decreases spasticity [6,7] and improves spinal reflex modulation [8], potentially by activating spinal inhibitory mechanisms [9]. Additionally, WBV has been associated with increased quadriceps strength [10] and improved walking ability [11], including walking speed [6]. Further, effects of WBV on walking ability and spasticity have been shown to persist into the week following a course of WBV [7,11]. However, effects of WBV have been shown to be dependent on dosage parameters, such as frequency and duration (i.e., number of bouts) [6].

Determining the optimal stimulation dose for improved walking function and decreased spasticity is crucial for translating WBV into clinical practice. Therefore, the purpose of the current study was to examine how different WBV doses affect walking speed, ankle control, and reflex excitability in participants with motor-incomplete SCI. Based on previous studies in which WBV increased CST descending drive to lower extremity muscles and the fact that CST has the strongest connection with ankle dorsiflexors during walking [3], it was expected that WBV would be associated with increased dorsiflexion during swing phase. Moreover, we predicted that the effects of WBV would be dose-dependent, such that participants in the long-bout group would have a greater increase in walking speed, an increase in dorsiflexion during swing phase, and a decrease in reflex excitability when compared with the short-bout group. We also assessed the associations between walking speed and ankle control, as measured by dorsiflexion during swing phase and plantar-flexor reflex excitability.

## 2. Materials and Methods

This study was carried out with ethics approval from the Shepherd Center Research Review Committee. All participants provided written informed consent prior to study enrollment in accordance with the Declaration of Helsinki, and the study was conducted in accordance with the Health Insurance Portability and Accountability Act guidelines. The current study is a supplemental analysis of data collected as part of a larger study focused on dose-response effects of WBV on spasticity in persons with SCI. This study was prospectively registered at clinicaltrials.gov (Identifier: NCT02340910).

### 2.1. Participants

Individuals were eligible for participation in the overall study if they met the following inclusion criteria: 16–72 years of age, ≥6 months since time of spinal cord injury, have at least mild spasticity affecting lower extremity muscles (as determined by participant self-report), able to sit at the edge of a mat without assistance of another person, and able to tolerate standing for at least 1 min. The current study analyzed data from participants who met the following additional inclusion criteria: the ability to walk 10 m without the use of an ankle–foot orthosis and presence of a measurable H-reflex during electrophysiological testing. Individuals were excluded for the following reasons: progressive or potentially progressive spinal lesions, neurological level of injury below spinal level T12, history of cardiovascular irregularities, problems following instructions, and orthopedic conditions that would limit their participation in the protocol (e.g., knee or hip flexion contractures > 10°).

### 2.2. Study Design

**Figure 1:**
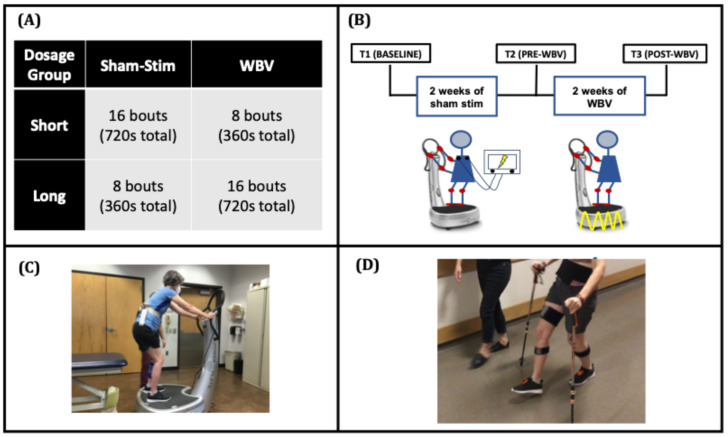
[Study Design] (**A**) Participants were stratified to receive short- or long-bout WBV. (**B**) The sham-stimulation wash-in phase (T1 to T2) consisted of 10 sessions of sham stimulation, in which participants stood on a WBV platform (**C**) for 45 s followed by 1 min of seated rest for the designated bouts set by the bout group. The whole-body vibration phase (T2 to T3) consisted of 9 sessions of 50 Hz WBV with the same stand/rest timing. The 10th WBV session occurred immediately after T3 testing. (**D**) Dorsiflexion during swing was captured during 10-m walk tests with inertial measurement units.

To assess the dose-response effects of WBV, we conducted a randomized, sham-control, wash-in study. A target sample of 16 participants was based on effects observed in an earlier multi-session study of WBV for quadriceps spasticity [7]. Baseline testing (T1) was followed by a 2-week sham-stimulation wash-in phase, pre-WBV testing (T2) followed by a 2-week WBV intervention phase, and finally, post-WBV testing (T3) (Figure 1).

During the sham-stimulation wash-in phase, all participants received 10 sessions of sham stimulation (every weekday for 2 weeks) while they performed repeated bouts of sit-to-stand activities. Each bout consisted of a 45-s period of standing on a WBV platform followed by a 1-min rest period. During the WBV intervention phase, participants received 9 sessions of WBV treatment over 2 weeks (with T3 before the 10th session). Participants were randomized to either short- or long-bout groups based on the number of bouts during the WBV intervention phase. Prior to enrolling the first participant, a random number generator was used to create an allocation sequence table based on simple randomization. The randomized allocation order was concealed from the study staff and was revealed to the staff performing the intervention only after baseline testing had been completed. The eight-bout stand/rest period replicates a prior single-session study of WBV wherein larger effects on quadriceps spasticity were observed with eight-bout sessions compared with effects observed with four-bout sessions [6]. In the present multi-session study, we sought to determine whether doubling the number of bouts from eight to sixteen would result in still larger effects. The number of bouts during the wash-in and intervention phases was counterbalanced. In this way, the short-bout group received sixteen bouts/sessions of sham stimulation followed by eight bouts/sessions of WBV, and the long-bout group received eight bouts/sessions of sham stimulation followed by 16 bouts/sessions of WBV. Walking and spasticity assessments were performed during each testing session.

### 2.3. Intervention

#### 2.3.1. Sham-Stimulation Wash-In Phase

During each sham-stimulation bout, participants stood on the WBV platform (Powerplate Pro 5, Performance Health Systems, LLC., Northbrook, IL, USA) for 45 s in a slight squat posture and were encouraged to use the handrails for balance only. Each bout was followed by 1 min of rest in a seated position.

The sham-stimulation intervention was provided by 2-inch round electrodes adhered to the posterior thoracic region (on the inferior angle of the scapula). The stimulator (Empi Continuum, DJO Global, Vista, CA, USA) was turned on, and intensity increased until participants indicated that they could feel the stimulation. Participants were then instructed that the stimulation would be reduced to an intensity that they could not feel, but in fact, the stimulator was turned off. While prior studies have suggested that there is no influence of stimulation when intensity is below sensory threshold [12,13], the rationale for turning the stimulation off completely was that in the presence of SCI, the lesion could prevent the participant from feeling the stimulation, such that intensities below sensory threshold could still be high enough to influence the excitability of spinal circuits.

#### 2.3.2. Whole-Body Vibration Phase

For the WBV phase of this study, participants received high-frequency (50 Hz) WBV. This frequency was chosen based on the results of a prior study indicating that 50 Hz has a larger antispasmodic effect compared with 30 Hz in persons with SCI [6]. The WBV bout consisted of standing on the WBV platform in the same posture and for the same duration/intervals as described for the sham condition. After each bout of vibration, participants rested in a seated position for 1 min.

### 2.4. Outcome Measures

All tests were performed on the more spastic leg as determined by the participant’s response to the query, “which of your legs is more spastic?”

#### 2.4.1. Walking Assessments

The 10 Meter Walk Test (10MWT) was performed to assess walking speed and kinematic data (i.e., dorsiflexion during the swing phase). Participants followed the standardized instruction: “walk as quickly and safely as possible” and walked a total distance of 14 m with 2 m before and after the middle 10 m walkway to allow for the assessment of a consistent gait pattern acceleration and deceleration. Participants used the assistive device they typically utilize in their daily lives and were instructed to use the same assistive device and wear the same pair of shoes for all trials throughout the study. A physical therapist ensured the participants’ safety during testing by using a gait belt. A hand-held stopwatch was used to record the time taken by participants to complete the 10MWT. Three trials were performed per session, with at least 2 min of rest in between. Mean walking speed across trials at each testing session was calculated as meters per second and used for subsequent analysis. Kinematic data were captured using 17 inertial measurement units (IMUs; Xsens MVN Awinda, Xsens Technologies B.V., Enschede, The Netherlands) that were attached at the following body segments: head, sternum, and pelvis, and bilaterally shoulders, upper arms, forearms, hands, thighs, shanks, and feet, in accordance with the manufacturer guidelines (MVN User Manual 2020, Xsens Technologies). All kinematic data was captured using a 60 Hz sampling frequency. For each walking trial, the IMU data were processed offline using the associated software (Xsens MVN version 2019.0.0), and the peak sagittal ankle angle values at mid-swing were extracted from the middle 50% of the step cycles of the more spastic leg, where positive angles indicated dorsiflexion and negative ankle angles indicated plantarflexion. Averaged ankle angle values from all trials at each testing session were used in the analysis.

#### 2.4.2. Spasticity Assessment

Modulation of spinal reflex excitability was assessed based on low-frequency depression of the soleus H-reflex. Low-frequency depression is a form of rate-dependent depression wherein repeated stimuli activate presynaptic inhibitory mechanisms that reduce reflex excitability. These inhibitory influences have been shown to be impaired in persons with SCI, which is thought to contribute to spasticity [5,14,15,16]. Briefly, participants were positioned reclined on a padded mat with legs extended and 2 wedges providing back support. Stimulating electrodes were placed over the tibial nerve in the popliteal fossa (cathode) and over the patella (anode). To record electromyographic data, bipolar electrodes (AgCl; Motion Lab Systems, Baton Rouge, LA, USA) were placed over the soleus in the area corresponding to the central portion of the muscle of the most spastic leg [17]. Signals were captured at a 1000 Hz sampling rate (Spike CED, Cambridge Electronic Design, Cambridge, UK). An H-reflex recruitment curve was constructed for each participant based on standard procedures [5,14]. Based on the recruitment curve, the stimulation intensity at which the H-wave was 10–30% of the maximum M-wave was used to assess low-frequency depression [18]. For low-frequency depression data acquisition, each trial consisted of a test pulse to the posterior tibial nerve followed by a train of 10 conditioned stimuli at a 1-s interstimulus interval. At least 4 trials were averaged for each participant based on the criteria of having an H-wave at 10–30% of the maximum M-wave during the test pulse [18]. Soleus H-reflex peak-to-peak amplitude, mV, was measured, and low-frequency depression percentage was calculated as (100 − ((average of the 10 conditioned stimuli)/test stimulus)) × 100%. A higher percentage of low-frequency depression is indicative of increased inhibition in the plantar-flexor spinal reflex.

### 2.5. Data Analysis

Data were analyzed using SPSS (version 26–29; SPSS Inc., Chicago, IL, USA). For continuous measures, the mean and standard deviation were calculated using Excel and displayed as mean (standard deviation). For categorical measures, the median and range were displayed as median (range). To investigate the effects of WBV dose on walking speed, ankle control, and reflex excitability over time, separate 3 × 2 mixed model repeated measures ANOVA with time (T1, T2, T3) as the within-subjects factor and dose (short-bout group, long-bout group) as the between-subjects factor were performed. All repeated measures of analyses reported here employed a multivariate approach using Wilks’ criterion. Post hoc pairwise comparisons using the least significant difference method were performed if significant main effects were present.

We did not find any significant main effects of WBV dosage, so we pooled the data from short and long-bout groups together for the subsequent analyses. The overall change in walking speed, ankle control, and reflex excitability between the sham-stimulation wash-in and the WBV intervention phase was assessed using one-tailed paired *t*-tests. To examine changes within each phase (sham stimulation wash in phase: T1 to T2; WBV intervention phase: T2 to T3), we also employed one-tailed paired t-tests. In addition, as repeated performance of the sit-to-stand activity has been shown to influence spasticity in both persons with SCI and stroke [19], we assessed the overall effects of 4 weeks of sit-to-stand training and compared T1 to T3. The significance level was set at *p* < 0.05 for all analyses as we did not adjust for multiple comparisons to avoid increasing the likelihood of committing type II errors (false negatives) [20,21]. We also computed effect sizes, which are considered more informative for clinical interpretation of results than *p*-values [22,23]. Per rehabilitation research guidelines, an effect size (Cohen’s d) of 0.16 is considered small, 0.31 is moderate, and 0.61 is large [24,25]. For the IMU data, range of error of the measurement was considered to be 1 – 2.15 degrees [26,27]. Finally, Pearson’s correlation coefficients (*r*) were used to investigate the relationship between walking speed, ankle control, and reflex excitability. *R* coefficients between 0.1 and 0.3 indicate weak relationships, between 0.3 and 0.5 moderate relationships, and greater than 0.5 strong relationships [25].

## 3. Results

### 3.1. Demographics

Sixteen participants (six female) from the larger study were able to walk 10 m without the use of an ankle–foot orthosis and had a measurable H-reflex, thereby meeting the inclusion criteria for this analysis (Table 1). The mean age was 51.13 (14.57) years, with an average time of 6.24 (8.23) years since injury. All participants were classified as having an American Spinal Injury Association (ASIA) impairment scale (AIS) score of AIS D. Neurological level of injury varied from C2 to T8. The median total lower extremity motor score, as measured from both legs, was 45 (range: 26–50) out of 50, with a median of 21 (range: 9–25) for the weaker side and 24 (range: 14–25) for the stronger side. Eight participants were randomized into the short-bout group, while the other eight participants were randomized into the long-bout group.

### 3.2. Effects of WBV Dose on Outcome Measures

#### 3.2.1. Walking Speed

The main effect of test time was significant (F (2,13) = 4.71, *p* = 0.03, partial η^2^ = 0.68), but there was not a significant main effect for WBV dose (F (1,14) = 1.39, *p* = 0.26, partial η^2^ = 0.09) or dose and time interaction (F (2,13) = 0.24, *p* = 0.79, partial η^2^ = 0.08) on walking speed. Post hoc analysis revealed that walking speed was significantly improved following the completion of WBV training compared with baseline.

#### 3.2.2. Ankle Control

There was not a significant main effect of WBV dose (F (1,14) = 1.46, *p* = 0.89, partial η^2^ = 0.02) and test time (F (2,13) = 0.06, *p* = 0.95, partial η^2^ = 0.01) on ankle dorsiflexion during the swing phase. No dose and time interaction on ankle control was observed (F (2,13) = 0.32, *p* = 0.73, partial η^2^ = 0.05).

#### 3.2.3. Reflex Excitability

There was not a significant main effect of WBV dose (F (1,14) = 0.001, *p* = 0.98, partial η^2^ = 0.00), test time (F (2,13) = 0.08, *p* = 0.92, partial η^2^ = 0.01), nor dose and test time interaction (F (2,13) = 0.02, *p* = 0.98, partial η^2^ = 0.003) on low-frequency depression.

### 3.3. Between-And Within-Phase Effects of WBV on Outcome Measures

There were no differences between the short- and long-bout WBV groups; therefore, the group data were pooled for all subsequent analyses to allow for between- and within-phase comparisons. Table 2 presents differences in walking speed, ankle control, and reflex excitability during the wash-in phase, intervention phase, and overall 4-week study period.

#### 3.3.1. Walking Speed (Figure 2)

Our findings did not demonstrate a significant change in walking speed between the sham stimulation wash-in and WBV intervention phases (*t*_15_ = 0.2, *p* = 0.42, d = 0.09). Within-phase differences indicated a significant change, 0.04 m/s (0.06), in walking speed when participants received WBV (*t*_15_ = 2.38, *p* = 0.02, d = 0.13) but not when they received sham stimulation (*t*_15_ = 1.48, *p* = 0.08, d = 0.15). We also found a significant increase, 0.08 m/s (0.11), in walking speed when we investigated the overall effects of 4 weeks of sit-to-stand training (*t*_15_ = 2.78, *p* = 0.007) with a small effect size (d = 0.28).

**Figure 2:**
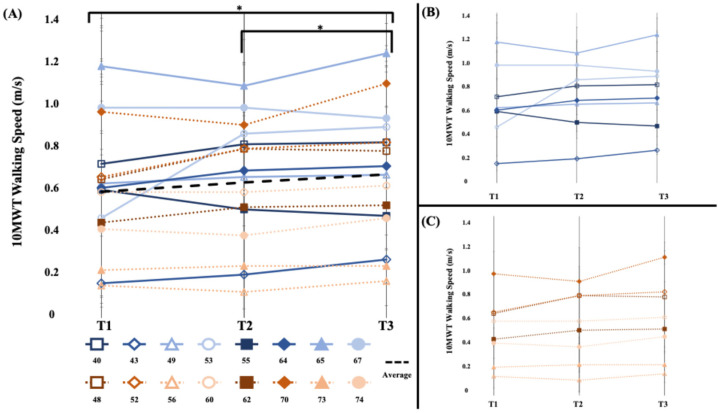
[10MWT Walking Speed (m/s)] (**A**) The 10-m walking test speed of the short- and long-bout groups. The average walking speed during the intervention phase (T2 to T3) and overall (T1 to T3) significantly improved. (**B**) Short-bout group, blue (**C**) Long-bout group, orange. Error bars represent standard error of mean across walks. Asterisk (*) indicates statistically significant changes at *p* < 0.05.

#### 3.3.2. Ankle Control (Figure 3)

There were no significant between-phase differences in dorsiflexion angle during the swing phase (*t*_15_ = 0.34, *p* = 0.37, d = 0.15). Within-phase comparisons demonstrated similar findings for both the sham stimulation wash-in phase (*t*_15_ = −0.34, *p* = 0.37, d = 0.07) and the WBV intervention phase (*t*_15_ = 0.24, *p* = 0.41, d = 0.04). Overall, the −0.12 (3.7) degree change in dorsiflexion during the 4-week study period was not significant (*t*_15_ = −0.13, *p* = 0.45, d = 0.02). In all cases, all group differences were less than the range of error of the measurement 1–2.15 degrees [26,27].

**Figure 3:**
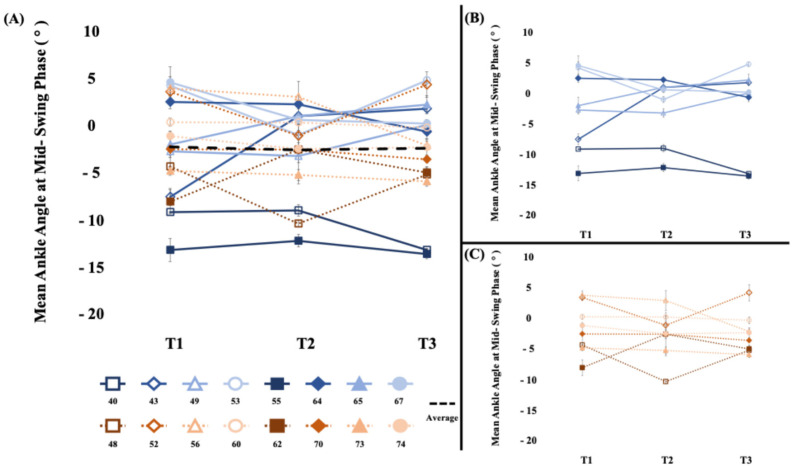
[Dorsiflexion during the swing phase (°)] (**A**) The average mean ankle angle at mid-swing phase of the short- and long-bout groups. There was not a significant change in dorsiflexion during the swing phase. (**B**) Short-bout group, blue (**C**) Long-bout group, orange. Positive values represent dorsiflexion, and negative values represent plantarflexion. Error bars represent standard error of mean across walks.

#### 3.3.3. Reflex Excitability (Figure 4)

Between-phase comparisons indicated that there were no differences in the low-frequency depression change between the sham stimulation wash-in and WBV intervention phases (*t*_15_ = 0.17, *p* = 0.43, d = 0.08; Table 2). Within-phase analyses of low-frequency depression revealed that this measure did not significantly change during the wash-in phase (*t*_15_ = −0.33, *p* = 0.75, d = 0.09), the intervention phase (*t*_15_ = −0.02, *p* = 0.98, d = 0.01), or the overall 4-week study period (*t*_15_ = −0.41, *p* = 0.34, d = 0.11).

**Figure 4:**
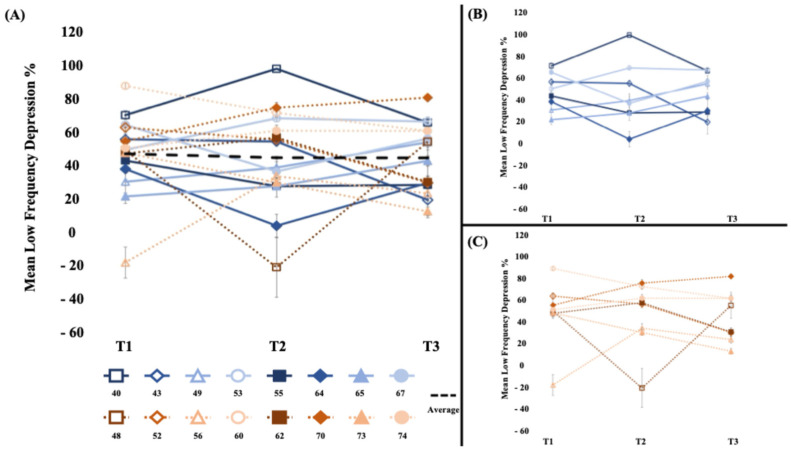
[Low-frequency depression %] (**A**) The average low-frequency depression % (100 − ((average of the 10 conditioned stimuli)/test stimulus)) × 100% of the short- and long-bout groups. There was not a significant change in low-frequency depression. (**B**) Short-bout group, blue (**C**) Long-bout group, orange. Higher percentage values indicate greater inhibition. Error bars represent standard error of mean.

### 3.4. Relationships Between Walking Speed, Ankle Control, and Low-Frequency Depression

Pearson’s correlation coefficients, presented in Table 3, were computed to investigate the relationship between walking speed, ankle control, and low-frequency depression. A moderate negative relationship between the change in walking speed during the wash-in phase and the change in dorsiflexion angle during the wash-in phase (*r* = −0.43, *p* = 0.05) was revealed. There were no other significant correlations during the same phase (Table 3). There were no significant correlations between overall changes in walking speed and dorsiflexion angle (*r* = 0.21, *p* = 0.22) or walking speed and low-frequency depression (*r* = 0.14, *p* = 0.31) during the study (Table 3).

## 4. Discussion

In this study, the objective was to determine if there were effects of different doses of robust noninvasive afferent stimulation, in the form of WBV, on walking speed, ankle control—as measured by dorsiflexion during the swing phase of gait—and plantar flexor reflex excitability in persons with SCI. Despite prior evidence that doubling the dose of WBV from four bouts to eight bouts resulted in a more robust effect on quadriceps spasticity [6], doubling the dose from eight bouts to sixteen bouts did not result in larger effects on any of the measures of interest. It is possible that, just as is observed with ceiling effects in pharmaceutical studies [28], there is an optimal dose of WBV, whereafter increasing the dose beyond this amount does not result in greater effectiveness.

Contrary to previous evidence that a single session of WBV decreased soleus spinal reflex circuit excitability [29] and the effect of WBV on walking outcomes [11], there was no impact of the dose of WBV on any of the outcome measures of interest. However, our results demonstrated that 2 weeks of sham stimulation followed by 2 weeks of WBV, regardless of the dose, significantly increased walking speed in individuals with SCI. We also observed small but insignificant gains in dorsiflexion during the swing phase. Overall, the current study is consistent with previous evidence that WBV may improve walking speed but cannot conclusively state whether the mechanism of the observed improvement is dose-specific or via improved dorsiflexor control.

### 4.1. Walking Speed and Ankle Control

Walking speed had significant improvement during the intervention phase but not during the wash-in phase. These results are in line with previous evidence that WBV improves walking speed [6,11]. Although walking speed did not reach a clinically meaningful important difference of 0.06 m/s after WBV [30], it could be due to the duration of the study. In the Musselman 2014 study [30], participants exhibited this change in walking speed after 3 to 4 months of body weight-supported treadmill training. Perhaps a future study period of whole-body vibration beyond 2 weeks could reach this change in walking speed. The mechanism by which WBV improves walking speed may be through the corticospinal descending drive, as a previous study demonstrated that motor-evoked potentials immediately increased after WBV [29]. Additionally, there is evidence that targeting corticospinal drive through other forms, such as anodal transcranial direct current stimulation, can improve walking speed [31]. However, future studies would benefit from exploring the timing of increased corticospinal drive through WBV or other means with increased walking speed.

In this analysis, an influence of WBV on ankle dorsiflexion during walking having a dose-related response magnitude was not identified. Neither hypothesis was supported by the data; differences were small and within the range of measurement error. A previous report of the effect of WBV on walking function in participants with SCI identified changes throughout the week following the last intervention. In that study, participants received three sessions per week of WBV for 4 weeks for a total of 12 sessions [11]. The changes in walking speed observed in the prior study may have been unrelated to improvements in dorsiflexion during the swing phase. Alternatively, while the cumulative dose of WBV in the current study was similar to that in the prior study, the absence of significant effects of WBV on ankle control in this study may be due to the distribution of the WBV dose, which was distributed over a longer time period in the prior study.

### 4.2. Reflex Modulation

The WBV intervention was not associated with significant differences in soleus H-reflex modulation as measured by low-frequency depression. A previous study in which a single session of WBV decreased spinal reflex excitability in the soleus [29] was the basis for the hypothesis that WBV would improve soleus spinal reflex modulation in persons with spasticity. Moreover, a prior study of participants with motor-incomplete SCI who received a 12-session WBV intervention over 4 weeks found that reductions in quadriceps excitability in response to stretch (the pendulum test) persisted 6–8 days after the final WBV intervention in some participants [7]. As with dorsiflexion, it is possible that the distribution of the WBV dose over a shorter time period was insufficient to evoke a neuroplastic effect.

### 4.3. Limitations

The study sample size of 16 participants may have been insufficient to observe effects even if present. Mean expected change in measures such as dorsiflexion angle, and LFD is small, and in the presence of large inter-individual differences in these measures, it can be difficult to detect change even if present (i.e., a type II error). Moreover, the homogeneity of the sample, with all participants classified as AIS D, limits generalizability and restricts the applicability of results to individuals with different SCI severity classifications. Future studies sufficiently powered to detect between- and within-phase differences will provide better insights into the effects of WBV on ankle control walking speed and reflex excitability.

While our key focus was on examining the dose-related effects of WBV, such effects were not identified. Further, while the wash-in period was intended to control for dose-related effects of movement-related activity (i.e., sit-to-stand), in the absence of dose-related effects of WBV, the direct effect of WBV on the outcomes observed cannot be definitively confirmed.

This study used IMUs for kinematic data collection, which may have a larger measurement error than optical motion capture systems. For this reason, differences in ankle angle were within the range of measurement error, and these results may be equivocal.

Finally, the study’s investigation into the mechanisms underlying WBV effects focused on assessing changes in dorsiflexion during the swing phase of gait and low-frequency depression of the H-reflex. While these provide valuable insights, there may be opportunities in future research to explore additional neurophysiological mechanisms, like examining changes in corticospinal excitability that may potentially play a significant role.

## 5. Conclusions

In conclusion, whole-body vibration does not have a dose-dependent impact on walking speed, ankle control, or reflex excitability. There is evidence that WBV does improve walking speed, but the current study cannot associate improved dorsiflexor control or plantar flexor reflex excitability to this outcome. In future studies, it will be important to assess CST descending drive and the association of dorsiflexor control with improved walking speed.

## Figures and Tables

**Table 1:** Demographics.

Subject ID	Sex	Age (Years)	Time Since Injury y (m)	AIS	Neurological Injury Level	LEMS (Weaker)	LEMS (Stronger)	LEMS (Total)	Spastic Leg	Bout Group
40	F	21	1 (0)	D	T11	24 (R)	25	49	R	Short
43	M	49	29 (5)	D	C5	18 (R)	25	43	R	Short
49	F	63	5 (8)	D	C5	21 (R)	24	45	R	Short
53	M	45	1 (9)	D	C4	9 (L)	17	26	L	Short
55	M	44	19 (11)	D	C2	11 (L)	25	36	L	Short
64	M	69	2 (1)	D	C4	21 (R)	24	45	R	Short
65	M	53	0 (6)	D	C3	25 (L)	25	50	L	Short
67	M	60	3 (3)	D	C5	23 (L)	24	47	L	Short
48	M	52	3 (4)	D	C4	23 (L)	23	46	L	Long
52	M	61	2 (0)	D	C1	23 (L)	25	48	L	Long
56	M	37	2 (6)	D	C3	13 (R)	14	27	L	Long
60	F	29	0 (11)	D	T8	22 (R)	22	44	R	Long
62	M	40	0 (8)	D	C4	21 (L)	25	46	L	Long
70	F	59	4 (8)	D	C7	24 (R)	25	49	R	Long
73	F	75	6 (11)	D	C6	14 (R)	23	37	R	Long
74	F	61	15 (3)	D	C5	21 (L)	24	45	L	Long

Abbreviations: AIS, American Spinal Injury Association Impairment Scale (D: Motor function is preserved below the neurological level); LEMS, lower extremity motor score; F, female; M, male; R, right; L, left.

**Table 2:** Sham-stimulation vs. WBV phase.

	Dorsiflexion During Swing ° Δ			Low-Frequency Depression % Δ			10MWT Walking Speed m/s Δ		
Subject ID	Sham	WBV	Overall	Sham	WBV	Overall	Sham	WBV	Overall
Short-Bout Group									
40	0.17	−4.13	−3.96	27.85	−32.35	−4.50	0.09	0.01	0.09
43	8.45	0.78	9.23	−1.31	−35.09	−36.41	0.04	0.08	0.11
49	−0.5	3.25	2.75	8.54	15.3	23.84	0.04	0.00	0.04
53	−5.16	5.74	0.58	18.82	−1.89	16.93	0.40	0.03	0.42
55	0.96	−1.38	−0.42	−15.38	0.75	−14.64	−0.09	−0.03	−0.12
64	−0.24	−2.86	−3.09	−34.03	25.69	−8.35	0.08	0.01	0.09
65	2.98	1.21	4.19	6.04	15.22	21.26	−0.09	0.14	0.06
67	−3.96	−0.39	−4.35	−28.07	19.82	−8.25	0.00	−0.05	−0.05
Long-Bout Group									
48	−6	5.15	−0.85	−70.26	75.41	5.15	0.15	−0.01	0.13
52	−4.58	5.35	0.77	−7.22	−25.92	−33.14	0.13	0.04	0.17
56	−0.87	−5.11	−5.97	51.74	−10.31	41.43	−0.03	0.04	0.01
60	−0.04	−0.54	−0.58	−16.24	−10.91	−27.16	−0.01	0.03	0.03
62	5.47	−2.42	3.05	9.49	−26.67	−17.18	0.07	0.01	0.07
70	−0.03	−1.03	−1.06	20.15	6.12	26.27	−0.06	0.19	0.13
73	−0.42	−0.68	−1.10	−17.9	−17.27	−35.17	0.02	0.01	0.03
74	−1.35	0.18	−1.17	10.44	−0.15	10.29	−0.04	0.08	0.05

Abbreviations: Sham, sham-stimulation wash-in phase; WBV, whole-body vibration phase; overall, change from T1 to T3; 10MWT, ten-meter walk test, m/s, meters per second; Δ, Change score.

**Table 3:** (**a**) Correlations by Phase; (**b**) Correlations Overall.

(**a**)												
	**DF Δ Sham Phase**		**DF Δ WBV Phase**		**LFD Δ Sham Phase**		**LFD Δ WBV Phase**		**Speed Δ Sham Phase**		**Speed Δ WBV Phase**	
	***r***	***p***	***r***	***p***	***r***	***p***	***r***	***p***	***r***	***p***	***r***	***p***
DF Δ Sham Phase			−0.45	0.04 *	0.29	0.14	−0.52	0.02 *	−0.43	0.05 *	0.31	0.12
DF Δ WBV Phase	−0.45	0.04 *			−0.37	0.08	0.34	1.00	0.55	0.01 *	0.00	0.50
LFD Δ Sham Phase	0.29	0.14	−0.37	0.08			−0.63	0.01 *	−0.08	0.38	0.42	0.05 *
LFD Δ WBV Phase	−0.52	0.02 *	0.34	1.00	−0.63	0.01 *			0.04	0.44	−0.14	0.30
Speed Δ Sham Phase	−0.43	0.05 *	0.55	0.01 *	−0.08	0.38	0.04	0.44			−0.27	0.15
Speed Δ WBV Phase	0.31	0.12	0.00	0.50	0.42	0.05 *	−0.14	0.30	−0.27	0.15		
(**b**)												
	**DF Δ Overall**		**LFD Δ Overall**		**Speed Δ Overall**							
	***r***	***p***	***r***	***p***	***r***	***p***						
DF Δ Overall			−0.30	0.13	0.21	0.22						
LFD Δ Overall	−0.30	0.13			0.14	0.31						
Speed Δ Overall	0.21	0.22	0.14	0.31								

Abbreviations: Sham phase, sham-stimulation wash-in phase; WBV phase, whole-body vibration phase; overall, across phases; DF, dorsiflexion during the swing phase; LFD, low-frequency depression; Speed, walking speed during ten-meter walk test. * Significant values *p* < 0.05, Pearson’s correlation test.

## Data Availability

Data are available upon written request.

## Abbreviations

The following abbreviations are used in this manuscript:

SCI	Spinal Cord Injury
WBV	Whole-body Vibration
CST	Corticospinal Tract
TA	Tibialis Anterior
10MWT	10 Meter Walk Test
IMU	Inertial Measurement Unit
LFD	Low-Frequency Depression
ASIA	American Spinal Injury Association
AIS	ASIA Impairment Scale
LEMS	Lower Extremity Motor Score
